# Inferring protein function by domain context similarities in protein-protein interaction networks

**DOI:** 10.1186/1471-2105-10-395

**Published:** 2009-12-02

**Authors:** Song Zhang, Hu Chen, Ke Liu, Zhirong Sun

**Affiliations:** 1 MOE Key Laboratory of Bioinformatics, State Key Laboratory of Biomembrane and Membrane Biotechnology, Department of Biological Sciences and Biotechnology, Tsinghua University, Beijing 100084, PR China

## Abstract

**Background:**

Genome sequencing projects generate massive amounts of sequence data but there are still many proteins whose functions remain unknown. The availability of large scale protein-protein interaction data sets makes it possible to develop new function prediction methods based on protein-protein interaction (PPI) networks. Although several existing methods combine multiple information resources, there is no study that integrates protein domain information and PPI networks to predict protein functions.

**Results:**

The domain context similarity can be a useful index to predict protein function similarity. The prediction accuracy of our method in yeast is between 63%-67%, which outperforms the other methods in terms of ROC curves.

**Conclusion:**

This paper presents a novel protein function prediction method that combines protein domain composition information and PPI networks. Performance evaluations show that this method outperforms existing methods.

## Background

Genome sequencing projects are generating massive amounts of sequence data, and the functional annotation of these sequences became one of the most challenging tasks, especially for the many proteins whose functions remain unknown. Traditional computational methods have utilized sequence features and machine learning algorithms to predict functions. In recent years, high-throughput technologies, such as yeast-two hybrid, have provided large scale protein-protein interaction data, making it possible to develop new function prediction methods based on protein-protein interaction (PPI) networks [1,2].

Existing protein function prediction methods based on PPI can be categorized into two classes: direct methods based only on the protein interactions and module-assisted methods [3]. Direct methods directly infer protein functions from interactions in the PPI networks while module-assisted methods first try to find functional modules in the PPI networks and then assign protein functions based on the module functions.

Direct methods are based on the assumption that interacting proteins probably have identical or similar functions [4-7]. This assumption is supported by previous studies which show that 70%-80% of proteins share at least one identical function with their interacting partners. Schwikowski et al [8] used a neighbor counting method to predict protein functions. They took up to three most frequent functions of interacting partners as indicators of the function of each protein, which turned out to cover over 70% of the known functions. Hisigaki [9] et al tried to predict protein functions by computing the Chi-square statistics as an indicator of functions that were statistically significantly frequent among neighboring proteins. Chua et al [10] investigated the relationships between functional similarity and network distance. They utilized functional information from proteins within 1 or 2 neighborhoods of a protein by giving different weights to different network distances.

Vazquez et al [11] assigned functions to proteins via an iterative algorithm by maximizing the number of edges that connect proteins with the same function. Other graph-based methods include those of Karaoz et al [6] and Nabieva et al [7].

Instead of predicting individual protein functions, module-assisted methods first identify functional modules in PPI networks and then assign functions to the proteins according to functions of the module members. These methods are based on previous observations that a group of cellular components and their interactions usually can be attributed to a specific function [3,12,13]. The approaches of different module-assisted methods vary mainly on the methods for identifying functional modules, which divide the methods into those based on network topology only and those which integrate multiple data sources. Network topology based methods include MCODE [13], a module-assisted method based on clustering coefficients, the clustering method of Rives et al [14] and the hierarchical clustering method of Spirin et al [15]. Ge et al [16] showed that proteins having similar functions tend to have similar expression patterns, which can be used to predict protein functions. Ideker et al [17] developed a framework to identify active sub-networks by detecting significant changes in expression over a particular set of conditions. Hanisch et al [18] applied a co-clustering methodology that combined similarities in gene expression patterns and network topologies. Hierarchical clustering was then used to define functional modules.

Although several existing methods have combined multiple information resources, such as gene expression information, gene regulatory networks and PPI networks, none of them have yet integrated protein domain information and PPI networks to predict protein functions. This paper presents a novel protein function prediction method that uses protein domain composition and PPI networks. This paper first demonstrates that proteins having similar functions are often in similar domain contexts in PPI networks and then develops the protein function prediction method based on this observation. The method gives satisfactory results compared to several existing methods.

## Methods

Yeast PPI network data was obtained from DIP database [19]. 4,389 proteins and 14,338 protein-protein interactions were included in the network. The yeast PPI network was chosen because it is comparatively more complete with fewer missing interactions. Nearly 70% of the 6,375 ORFs of yeast are covered by the yeast PPI network, which is the highest coverage ratio among PPI networks of all organisms. Besides, the yeast PPI network is the most frequently used in previous protein function prediction studies, which allows accuracy comparison to other methods.

The domain annotation information was retrieved from the PFAM database [20,21]. The HMMER software package was used to annotate domains in the yeast ORFs. 6,402 domains of 4,618 domain types were obtained from 3,901 proteins. The protein function annotation information was provided by the Gene Ontology database [22].

**Figure 1:**
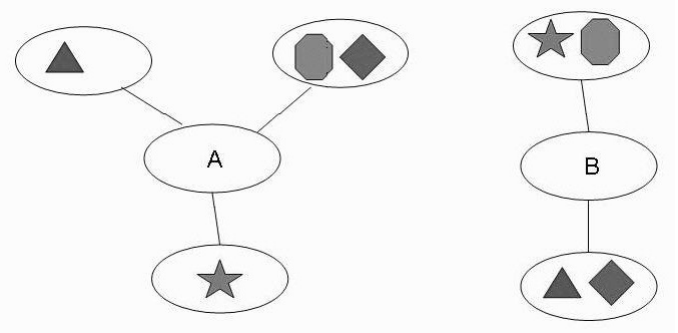
**Illustration of domain context similarity in PPI network**.

Domains are basic functional units in proteins. Cellular functions are accomplished by the cooperation of many domains in proteins. Therefore, the PPI network was decomposed into the domain level to investigate protein functions in terms of domain. Figure 1 shows a simplified model. Protein A has 3 neighbors in the PPI network, which all-together contain 4 different domains, while protein B has 2 neighbors with the same 4 domains. Domain shuffling or recombination during evolution may have changed the domain distribution among proteins. One possible situation is that domain 1 (represented as the diamond in Fig. 1) is in the same protein with domain 2 (represented as the octagon) in one organism, while in another organism, it is combined with domain 3 (represented as the triangle). Despite the different domain distribution, similar domain compositions of neighbors of protein A and B in the PPI network may indicate both functional similarity and evolutionary relationship. Thus proteins with similar domain contexts in the PPI network may share similar functions.

The domain context similarity (denoted as f) is defined as:

Where M is the number of domain types in the PPI network. Given proteins A and B, SA and SB are the sets of domains included in A's neighbors and in B's neighbors. The number of domain types in SA is a, while the number of domain types in SB is b. The intersection of SA and SB is S, containing s types of domains. C(M:s) denotes combinatorial numbers. The larger f indicates a greater probability that A and B share similar functions.

For each GO term, there is a positive data set composed of present proteins, and a negative data set including absent ones. For example, GO:0009277 is used to describe 107 yeast proteins, so these proteins were treated as positive samples. Since some GO terms contains only a few proteins and other GO terms are too general, only GO terms containing 10-200 proteins were considered.

Given a protein P with unknown function, in order to examine its function with regard to each particular GO term, the domain context similarities, f, between P and each protein in both the positive and negative data sets were calculated. The function annotation of the protein with the highest f value was then assigned to P.

The 7-fold cross validation, which has been widely implemented in previous researches [23,24], was used to evaluate the performance of our prediction. For every GO term, both the positive and negative data sets were divided into seven equal parts randomly. Every time six positive parts and six negative parts were used as the training data set while the remaining parts was used as the test data set. This procedure was repeated 7 times to ensure that every part was used once as the test data set for one GO term. Then the whole procedure was repeated for every GO term. The final accuracy was the average of the evaluations.

Four frequently used measurement indices, accuracy, precision, recall and Mathew correlation coefficient (MCC), were used to evaluate the prediction performance. The Mathew correlation coefficient (MCC) was calculated to assess the prediction performance when the numbers of proteins in the positive and negative data sets differed significantly. MCC ranges from -1 to 1, a larger MCC indicating better prediction performance. For data with positive predictions, the real positives are defined as true positives (TP), while others are defined as false positives (FP). For data with negative predictions, the real positives are defined as false negatives (FN), while the others are defined as true negatives (TN). Then, the measurement indices are defined as:

## Results

**Figure 2:**
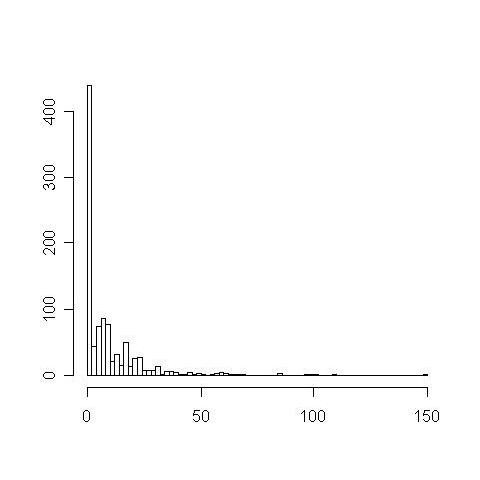
**f value distribution of set A**.

**Figure 3:**
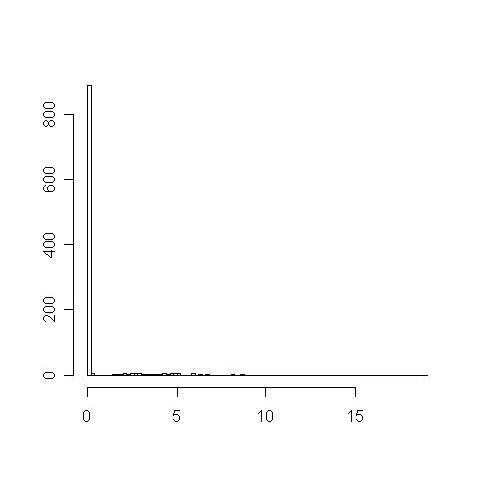
**f value distribution of set B**.

The relationships between protein function similarity and domain context similarity in the PPI network were investigated based on the measurement indices. First, 1000 pairs of proteins were randomly extracted from one GO term with the domain context similarity, f, then calculated for each pair (denoted as set A). Secondly, another 1000 random protein pairs were generated using pair of proteins from different GO terms. Their f values are also calculated (denoted as set B). The two sets of similarities were then compared to demonstrate the positive significant relationship between functional similarity and domain context similarity. The results showed that set A has a mean similarity, f, of 9.23 compared to 0.46 for set B. Kolmogorov-Smirnov test showed that set A is significantly higher than set B with a p-value less than 10^-15^. The distributions of the similarity for sets A and B are shown in Figures 2 and 3. There are many values between 0 and 150 in set A, while most values in set B are near 0. Hence, the domain context similarity can be a useful index to predict protein function similarity.

The method was then used to predict protein functions in yeast. GO terms were divided into 4 groups according to the number of proteins in each GO term. GO terms containing less than 10 proteins were excluded due to the lack of a satisfactory number of proteins for accurate predictions. GO terms including more than 200 proteins were also eliminated because the function annotations in these GO terms are usually too general. The results are shown in Table 1.

**Table 1:** Prediction performance measurements

Protein number in each GO term	GO term number	Accuracy	Precision	Recall	MCC
10-30	387	67.29%	64.73%	75.98%	0.43
30-50	81	66.71%	64.63%	73.83%	0.41
50-100	63	65.47%	65.60%	65.07%	0.37
100-200	8	63.06%	62.70%	64.46%	0.30
In total	539	66.29%	64.83%	71.18%	0.40

The prediction accuracies are between 63%-67%. The results show that the method has satisfactory robustness for various numbers of proteins within one GO term. As number of proteins increases from 10-30 to 100-200, the accuracy only decreases slightly, by about 4%. The phenomenon that accuracies decrease as number of proteins in the GO term increases can be attributed to the fact that functional annotations in larger GO terms are not as specific as in smaller GO terms. Fuzzy, general annotation information may affect the prediction performance. Further investigation is required to explain this observation. Besides, the recall is higher than the precision, demonstrating that false positive predictions are more common than false negative predictions.

**Figure 4:**
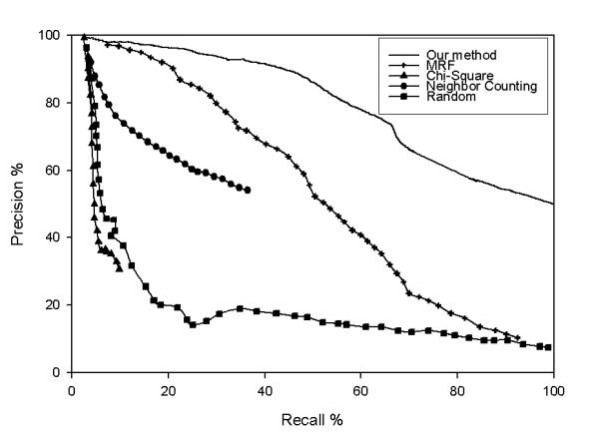
**ROC curves of several existing methods and our method**.

This method was then compared with existing methods based on the ROC curves. The three previously developed methods included in comparison are MRF [25], Neighbor counting [8] and Chi-square method [9]. The random prediction performance was also presented. The ROC curves shown in Figure 4 indicate that the current method outperforms the other methods.

## Discussion

A new prediction method for protein function based on protein-protein interaction and domain context was presented in this research. Domain context similarity in the protein-protein interaction network was defined and used as in index for prediction. The underling principle of this method was that proteins tend to interact with each other via domain-domain interaction. So the high quality domain-domain interaction information may improve the prediction accuracy. Riley at al [26] developed domain pair exclusion analysis (DPEA) to infer high-confidence domain interaction from protein interactions. Besides, DIMA [27,28] try to identify known and predicted domain interactions which may be helpful if this information was utilized in our method.

This research also suggests several future directions of research. First, domain context similarity measurements or prediction systems can be improved to reduce false positive predictions and boost accuracy. For example, the cutoff value for domain context similarity can be introduced to improve the accuracy and to deal with multiple function problems. Since the underlying rationale of this method is the domain-domain interaction, high-quality domain interactions can definitely contribute to the accuracy. As mentioned above, the newly developed domain interaction inferring method [26-28] can be used in our future algorithm improvement. Second, as shown by Chua et al [10], functional similarities exist between neighbor proteins with distances equal to or larger than 2, which may be useful information to be included in function prediction. Third, other data resources, such as gene expression profiles and gene regulatory networks, could be combined with domain context information to prediction functions. Different weight can be assigned to different types of information. Machine learning methods, such as SVM, can also be utilized to take the information listed above as input features. Finally, since protein domains are conserved and can be easily detected in various organisms, this method should be promising in comparing protein functions across species.

## Conclusion

The availability of large scale protein-protein interaction data sets makes it possible to predict protein functions based on protein-protein interaction (PPI) networks. Several existing methods combine multiple information resources to predict protein functions. We present a novel protein function prediction method that combines protein domain composition information and PPI networks. Performance evaluations show that this method outperforms existing methods. The results are used to analyze the relationships between domain context similarity and protein function similarity, while this research may have potential future research directions.

## Competing interests

The authors declare that they have no competing interests.

## Authors' contributions

ZS, HC designed the study, collected study data, performed the analysis, prediction and cross validation and produced the first draft of the manuscript. KL provided assistance in the process of collecting data and revising the manuscript. ZS was involved in designing the study and revising the manuscript. All authors read and approved the final manuscript.
